# Accuracy of an autocalibrated pulse contour analysis in cardiac surgery patients: a bi-center clinical trial

**DOI:** 10.1186/s12871-015-0153-2

**Published:** 2015-11-26

**Authors:** Ole Broch, Jose Carbonell, Carlos Ferrando, Malte Metzner, Arne Carstens, Martin Albrecht, Matthias Gruenewald, Jan Höcker, Marina Soro, Markus Steinfath, Jochen Renner, Berthold Bein

**Affiliations:** Department of Anaesthesiology and Intensive Care Medicine, University Hospital Schleswig-Holstein, Campus Kiel, Schwanenweg 21, D-24105 Kiel, Germany; Department of Anaesthesiology and Critical Care, University Hospital Valencia, Avenida Blasco Ibanez 17, 46010 Valencia, Spain; Christian-Albrechts-University Kiel, Schleswig-Holstein, Germany; Department of Anaesthesiology and Intensive Care Medicine, Asklepios Hospital St. Georg, Hamburg, Germany

**Keywords:** Cardiac index, Pulse contour analysis, Haemodynamic monitoring, Transpulmonary thermodilution

## Abstract

**Background:**

Less-invasive and easy to install monitoring systems for continuous estimation of cardiac index (CI) have gained increasing interest, especially in cardiac surgery patients who often exhibit abrupt haemodynamic changes. The aim of the present study was to compare the accuracy of CI by a new semi-invasive monitoring system with transpulmonary thermodilution before and after cardiopulmonary bypass (CPB).

**Methods:**

Sixty-five patients (41 Germany, 24 Spain) scheduled for elective coronary surgery were studied before and after CPB, respectively. Measurements included CI obtained by transpulmonary thermodilution (CI_TPTD_) and autocalibrated semi-invasive pulse contour analysis (CI_PFX_). Percentage changes of CI were also calculated.

**Results:**

There was only a poor correlation between CI_TPTD_ and CI_PFX_ both before (*r*^2^ = 0.34, *p* < 0.0001) and after (*r*^2^ = 0.31, *p* < 0.0001) CPB, with a percentage error (PE) of 62 and 49 %, respectively. Four quadrant plots revealed a concordance rate over 90 % indicating an acceptable correlation of trends between CI_TPTD_ and CI_PFX_ before (concordance: 93 %) and after (concordance: 94 %) CPB. In contrast, polar plot analysis showed poor trending before and an acceptable trending ability of changes in CI after CPB.

**Conclusions:**

Semi-invasive CI by autocalibrated pulse contour analysis showed a poor ability to estimate CI compared with transpulmonary thermodilution. Furthermore, the new semi-invasive device revealed an acceptable trending ability for haemodynamic changes only after CPB.

**Trial registration:**

ClinicalTrials.gov: NCT02312505 Date: 12.03.2012

## Background

Most of the studies applying algorithms for haemodynamic optimization of high-risk surgical patients have used cardiac index (CI) as one important target. Furthermore, these investigations could demonstrate that optimization of CI was associated with a significant lower rate of postoperative morbidity and mortality [1]. In the past, estimation of CI was mostly performed by pulmonary or transpulmonary thermodilution (TPTD) which due to their invasiveness are associated with considerable complications [2–4]. Therefore, interest has focused on less-invasive, readily available and easy to install techniques which are based for example on continuous arterial waveform analysis [5–7]. By using established arterial catheters, pulse contour analysis offers the opportunity for continuous estimation of CI and other haemodynamic variables like systemic vascular resistance or stroke volume variation, enabling the clinician to respond quickly and effectively to abrupt haemodynamic changes. The recently introduced semi-invasive monitoring system PulsioFlex (Pulsion Medical Systems, Munich, Germany) was developed for continuous CI trending and consists of an algorithm that provides beat-to-beat estimation of CI by analysis of the arterial blood pressure tracing. By using a proprietary “autocalibration” mode this software also calculates the individual aortic compliance and systemic vascular resistance based on patient data such as age, height, weight and gender.

The aim of the present study was to investigate accuracy and trending ability of the autocalibrated semi-invasive CI (CI_PFX_) with transpulmonary thermodilution (CI_TPTD_) before and after cardiopulmonary bypass (CPB).

## Methods

This study was conducted in compliance with the Helsinki declaration. After approval from institutional ethics committee (Ethikkomission UKSH Kiel - AZ 162/10, Christian-Albrechts-University Kiel, Schwanenweg 20, D 24105 Kiel; Comite Etico de Investigación Clinica, Hospital Clinico Universitario, Blasco Ibanez 17, Valencia 46010 Spain), written informed consent for participation in the study was obtained preoperatively from all patients. The trial was registered on ClinicalTrials.gov (NCT02312505). Sixty-five patients (41 patients Germany, 24 patients Spain) undergoing elective coronary artery bypass grafting (CABG) were studied after induction of general anaesthesia until discharge to the intensive care unit. Exclusion criteria were patients less than 18 years of age, a left ventricular ejection fraction ≤0.5, a lack of sinus rhythm, valvular heart diseases, emergency procedures and patients requiring mechanical support or continuous high-dose (>0.1 μg/kg/min) catecholamine therapy.

### Study protocol

All patients received midazolam 0.1 mg/kg orally 30 minutes before induction of anaesthesia. After establishment of monitoring of peripheral oxygen saturation (SpO_2_) and heart rate (HR) patients received a peripheral venous access and a radial arterial line in Seldinger-technique (Arrow International, Inc. Reading, PA, USA). According to the manufacturer’s instructions, a PulsioFlex system (Pulsion Medical Systems, Munich, Germany) was connected to the arterial line. Adjustment of the transducer was followed by zeroing and input of individual demographic data. Thereafter, autocalibration of the semi-invasive device was performed. All variables were automatically indexed to body surface area.

After induction of anaesthesia, a central venous catheter and a transpulmonary thermodilution catheter (Pulsion Medical Systems, Munich, Germany) were introduced in the right internal jugular vein and in the femoral artery, respectively. Patients were ventilated with the ADU S5 ventilator (Datex Ohmeda, GE Healthcare, Munich, Germany) in a volume-controlled mode with a tidal volume of 6–8 ml/kg, a positive end-expiratory pressure of 5 cm H_2_O, an I:E ratio of 1:1.5 and a FiO_2_ of 0.5. Respiratory rate was adjusted to achieve normocapnia (pCO_2_ 35–40 mmHg) and end-tidal carbon dioxid was measured with an infrared absorption analyzer. The thermodilution catheter was connected to the PiCCO_2_ monitor (Software version 1.3.0.8). The passive leg raising manoeuvre (PLR) was performed by a leg elevation up to 45° with the trunk in the horizontal position, inducing haemodynamic changes by transferring blood towards the central compartment.

### Data collection

**Fig. 1:**
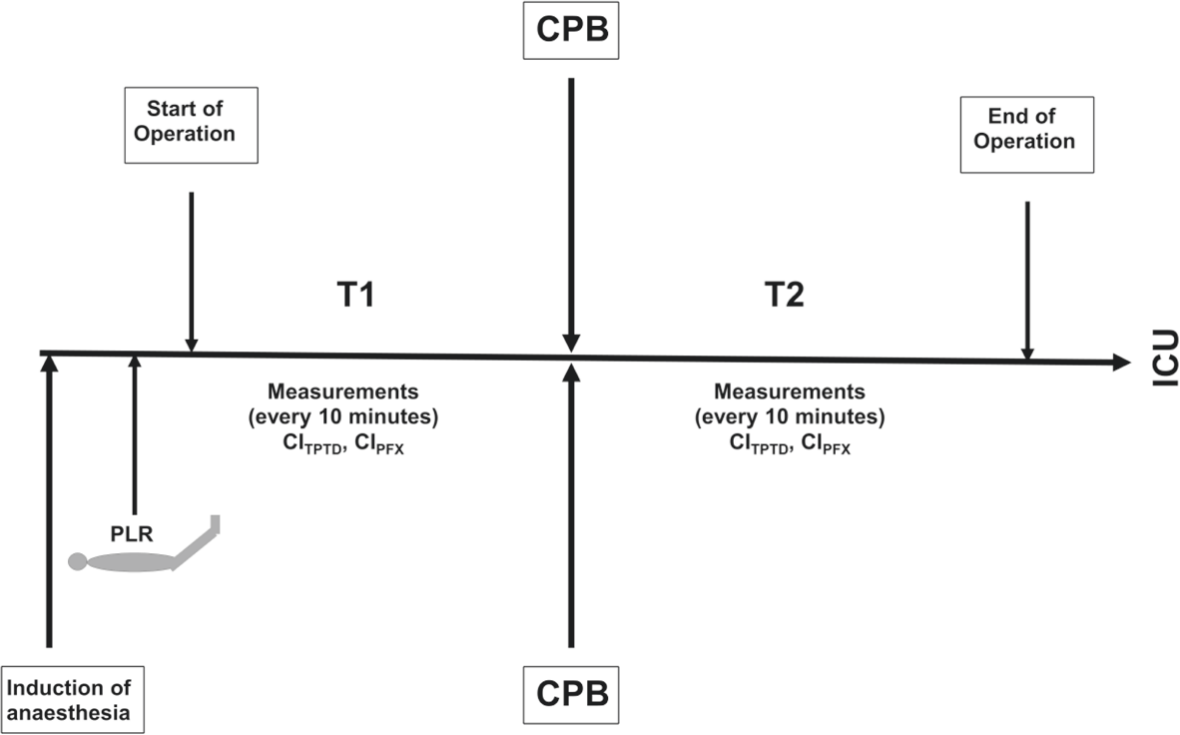
Study design with data collection starting after induction of anaesthesia and PLR until CPB (T1) and data collection restarting after CPB until the end of surgical intervention (T2); CI_TPTD_, cardiac index by transpulmonary thermodilution; CI_PFX_, cardiac index by semi-invasive pulse contour analysis; PLR, passive leg raising; CPB, cardiopulmonary bypass.

After induction of anaesthesia and establishment of all monitoring devices including autocalibration of the semi-invasive device, a PLR was performed and haemodynamic variables including CI (CI_TPTD,_ CI_PFX_) and stroke volume index (SVI) were recorded before, during and after PLR. Subsequently, estimation of CI_TPTD_ and CI_PFX_ were carried out every 10 minutes until the beginning of CPB (T1). Number of measurements differed from patient to patient, depending on the experience of the surgeon and time needed for preparation. Stable haemodynamic conditions and exclusion of an under- or overdamped arterial signal were prerequisites for the measurements. Estimation of CI_TPTD_ was based on injecting 15 ml ice cold saline (≤8°C) at least three times through the central venous line. Measurements were repeated if CI between individual measurements differed ≥15 %. Estimation of CI_PFX_ was performed simultaneously by recording and averaging five values over a period of two minutes. In case of a difference ≥15 % values of CI_PFX_ were also discarded. Fifteen minutes after weaning from CPB, autocalibration of the semi-invasive monitoring system was performed again and estimation of CI_TPTD_ and CI_PFX_ was restarted up to the end of the surgical intervention (T2). Again, due to different time needed for surgery, a different number of measurement pairs in individual patients were obtained during this time period (Fig. 1). There was no deviation from the study protocol.

### Statistical analysis

Statistical comparisons were performed using commercially available statistics software (GraphPad Prism 5, GraphPad Software Inc., San Diego, CA, USA; MedCalc for Windows, version 11.6.1.0, MedCalc Software, Mariakerke, Belgium; SigmaPlot 13.0 for Windows version 7, Systat Software, Inc., San Jose, CA). For demonstration of the relationship between sample size and the width of the confidence interval of the estimated variable, we calculated the width of the 95 % confidence interval of the limits of agreement (as $\pm 1.96\sqrt{\frac{3}{n}}\cdot s$, where s is the standard deviation of the bias) as recommended by Bland and Altman [8]. All data are given as mean ± SD and a p value <0.05 was considered significant. Linear correlations between the measurements of CI_TPTD_ and CI_PFX_ were calculated. To plot the agreement between CI_TPTD_ and CI_PFX_, a Bland-Altman analysis for repeated measurements was performed for each time period (T1-T2). We used the limits of agreement (2SD) of the bias divided by the mean CI values from CI_TPTD_ and CI_PFX_ for calculation of the percentage error and determined a 30 % threshold as previously described by Critchley and colleagues [9]. As suggested by a recent literature, we described the trending ability using different statistical techniques [10, 11]. Trending ability was assessed by determination of correlation coefficients between ∆CI_TPTD_ and ∆CI_PFX_, by a modified Bland-Altman analysis using the change in CI between sequential readings, a four quadrant analysis and polar plots. The concordance in the direction of change between ∆CI_TPTD_ and ∆CI_PFX_ was estimated. Changes of CI_TPTD_ < 15 % were excluded from analysis and a concordance rate of >90 % was considered reflecting a reliable trending ability as recommended by Critchley and colleagues [11]. The distance from the center of the polar plots reflects the mean change in CI. The angle ϴ with the horizontal axis represents agreement between the ∆CI value and reference technique (∆CI_TPTD_). The higher the agreement, the closer data pairs will lie along the radial axis. If ϴ equals 0°, the agreement between both ∆CI values is 100 %, but if ϴ is 90° there is no agreement at all. An unpaired sample t - test was used to analyse significant differences of arterial pressure and systemic vascular resistance index (SVRI) related to the periods of measurement.

## Results

Data of all 65 patients, 41 males and 24 females, were included into final analysis. Not a single patient suffered from any complication in the context of the present study. Age ranged between 39–81 years, with a mean age of 65 ± 3 years and a mean body mass index of 25.9 ± 2.8 kg/m^2^. Mean left ventricular ejection fraction was 0.62 ± 0.09. A total of 548 data pairs (T1: 288, T2: 260) were obtained during the study period. Unpaired t-test showed a significant difference (*p* < 0.05) between SVRI, heart rate and CI (CI_TPTD_, CI_PFX_) before (T1) and after CPB (T2). Haemodynamic variables are shown in Table 1.

**Table 1:** Haemodynamic variables before and after cardiopulmonary bypass

	pre - CPB	post - CPB	
Variables	T1	T2	*p*
	*n* = 288	*n* = 260	
HR (min^−1^)	55 ± 5	81 ± 3	*p* < 0.05^*^
MAP (mmHg)	75 ± 4	74 ± 7	*p* = 0.68
SAP (mmHg)	114 ± 15	112 ± 13	*p* = 0.08
DAP (mmHg)	54 ± 11	53 ± 9	*p* = 0.43
CVP (mmHg)	9 ± 3	10 ± 2	*p* = 0.11
SVRI_TPTD_ (dynes∙s/cm^5^/m^2^)	1820 ± 73	1472 ± 109	*p* < 0.05^*^
CI_TPTD_ (L/min/m^2^) max	5.0 ± 0.5	5.3 ± 0.8	*p* < 0.05^*^
CI_TPTD_ (L/min/m^2^) mean	2.2 ± 0.5	2.9 ± 0.7	*p* < 0.05^*^
CI_TPTD_ (L/min/m^2^) min	1.2 ± 0.3	1.5 ± 0.6	*p* < 0.05^*^
CI_PFX_ (L/min/m^2^) max	6.8 ± 0.6	7.0 ± 0.8	*p* < 0.05^*^
CI_PFX_ (L/min/m^2^) mean	2.8 ± 0.6	3.4 ± 0.5	*p* < 0.05^*^
CI_PFX_ (L/min/m^2^) min	1.1 ± 0.4	1.7 ± 0.7	*p* < 0.05^*^

*CPB* cardiopulmonary bypass, *HR* heart rate, *MAP* mean arterial pressure, *SAP* systolic arterial pressure, *DAP* diastolic arterial pressure, *CVP* central venous pressure, *SVRI*_*TPTD*_, systemic vascular resistance index measured by transpulmonary thermodilution *CI*_*TPTD*_, cardiac index by transpulmonary thermodilution, *CI*_*PFX*_ cardiac index by autocalibrated semi-invasive pulse contour analysis

Values are given as maximum, mean and minimum ± SD

^*^ *p* < 0.05 (vs. T1)

**Fig. 2:**
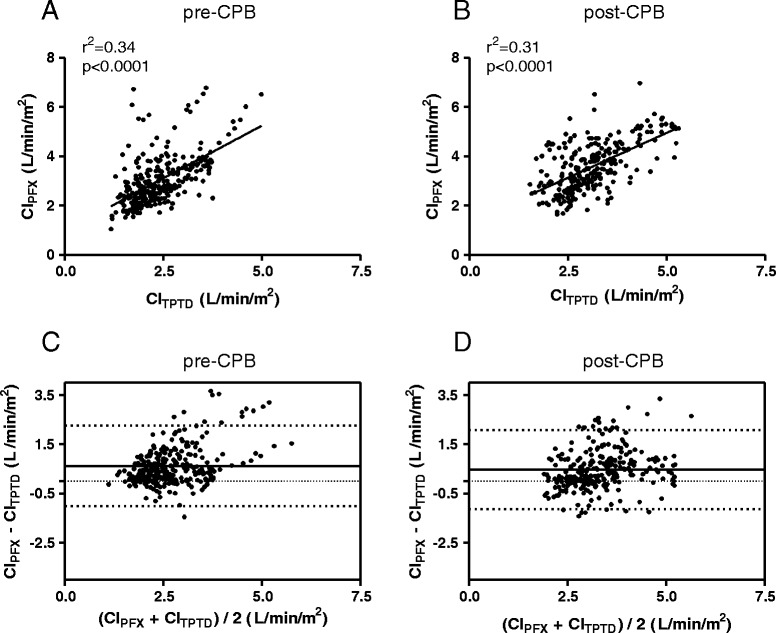
Before (pre) and after (post) cardiopulmonary bypass (CPB): **a**, **b** Correlation of cardiac index estimated by transpulmonary thermodilution (CI_TPTD_) and cardiac index estimated by semi-invasive autocalibrated pulse contour analysis (CI_PFX_); **c**, **d** Bland-Altman analysis showing the agreement between cardiac index estimated by transpulmonary thermodilution (CI_TPTD_) and cardiac index estimated by semi-invasive autocalibrated pulse contour analysis (CI_PFX_).

There was a moderate but significant correlation between CI_TPTD_ and CI_PFX_ at T1 (*r*^2^ = 0.34, *p* < 0.0001) and T2 (*r*^2^ = 0.31, *p* < 0.0001) (Fig. 2). Bland-Altman analysis for CI_TPTD_ and CI_PFX_ showed a mean bias of 0.65 L/min/m^2^, 95 % limits of agreement (LOAs) from −1.01 to +2.29 L/min/m^2^ before and a bias of 0.49 L/min/m^2^ with LOAs from −1.15 to +2.13 L/min/m^2^ after CPB. Percentage error (PE) was 63 % before and 50 % after CPB. Bias, LOAs and percentage errors for each time period (T1 - T2) are summarized in Table 2.

**Table 2:** Bland-Altman analysis showing bias, 95 % limits of agreement, confidence interval and percentage error before (T1) and after cardiopulmonary bypass (T2)

	T1	T2
ndata/npatient	*n* = 288/*n* = 65	*n* = 260/*n* = 65
	CI_PFX_	CI_PFX_
Mean (L/min/m^2^)	3.03	3.58
Bias (L/min/m^2^)	0.65	0.49
SD of bias (L/min/m^2^)	0.81	0.80
Confidence Interval of LOA (L/min/m^2^)	0.52	0.49
95 % limits of agreement (L/min/m^2^)	−1.01 to +2.29	−1.15 to +2.13
Percentage error (%)	63	50

*CI*_*PFX*_ cardiac index by semi-invasive pulse contour analysis, *CI of LOA* confidence interval of the limits of agreement

Values are given as mean ± SD

**Fig. 3:**
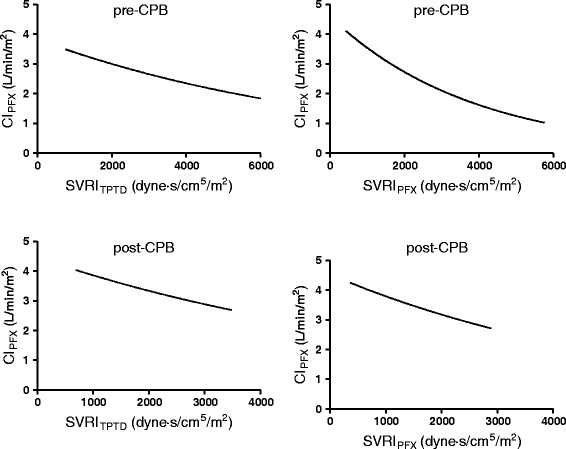
Exponential function of correlation between cardiac index measured by autocalibrated semi-invasive pulse contour analysis (CI_PFX_) and systemic vascular resistance index (SVRI) estimated by transpulmonary thermodilution (SVRI_TPTD_) and by pulse contour analysis (SVRI_PFX_) before (pre) and after (post) cardiopulmonary bypass (CPB)

There was a weak correlation between CI_PFX_ and SVRI determined by transpulmonary thermodilution (SVRI_TPTD_) and by pulse contour analysis (SVRI_PFX_) for both time periods (Fig. 3).

A PLR-manoeuvre before CPB was performed in all 65 patients. Responders increased their SVI_TPTD_ >15 % during PLR. We observed 37 responders (57 %) and there was a moderate correlation between CI_TPTD_ and CI_PFX_ (*r*^2^ = 0.28, *p* < 0.0001). During PLR, Bland Altman analysis showed a mean bias of 0.49 L/min/m^2^ and LOAs from −2.01 to +1.02 L/min/m^2^ with a percentage error of 68 % for CI_PFX_.

**Fig. 4:**
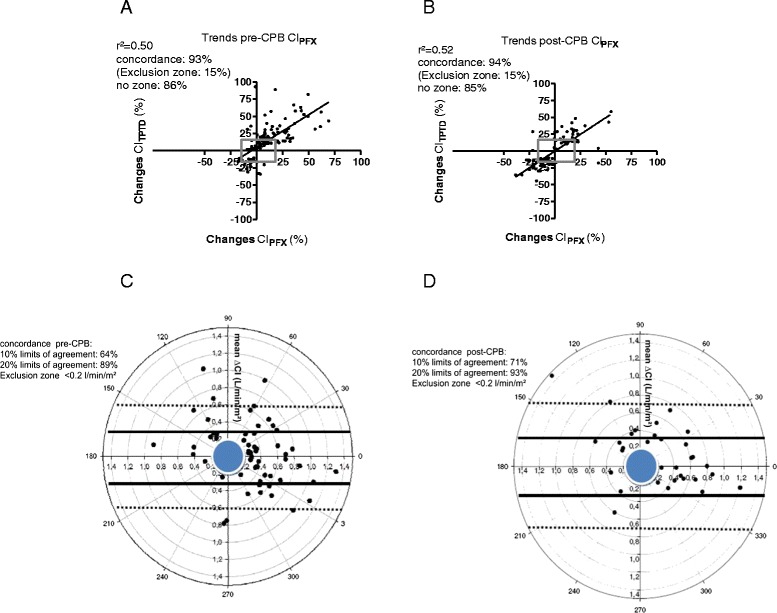
**a**, **b** Four quadrant concordance plots of changes of cardiac index measured by transpulmonary thermodilution (CI_TPTD_) and cardiac index estimated by autocalibrated semi-invasive pulse contour analysis (CI_PFX_) before and after cardiopulmonary bypass (CPB). Changes below 15 % (gray rectangle) were excluded from correlation analysis. **c**, **d** Polar plot analysis on trending ability of changes in cardiac index (∆CI) before and after cardiopulmonary bypass (CPB). The solid line included data pairs within the 10 % limits of agreement (± 0.30 L/min/m^2^ before and ± 0.35 L/min/m^2^ after CPB) and indicated good trending. Data pairs within the 20 % limits of agreement (±0.6 L/min/m^2^ before and ± 0.7 L/min/m^2^ after CPB, dotted line) indicated acceptable trending ability. The mean CI was 3.0 L/min/m^2^ before and 3.5 L/min/m^2^ after CPB. Exclusion zone was determined <0.2 L/min/m^2^.

After exclusion of percentage changes <15 %, correlation coefficients between ∆CI_TPTD_ and ∆CI_PFX_ were *r*^2^ = 0.50, *p* < 0.0001 before and *r*^2^ = 0.52, *p* < 0.0001 after CPB. Modified Bland-Altman analysis showed a bias of −4 %, with LOAs from −42 % to +33 % before CPB and of 0.18 % with LOA`s from −28 % to +29 % after CPB, respectively. Four quadrant plots revealed a concordance rate over 90 % indicating an acceptable ability for reflecting haemodynamic changes before and after CPB (Fig. 4a, b). Polar plot analysis demonstrated a poor trending ability before CPB (data within 10 % limits of agreement: 64 %, 20 % limits of agreement: 89 %) and an acceptable trending ability after CPB (data within 10 % limits of agreement: 71 %, 20 % limits of agreement: 93 %) for mean ∆CI_PFX_ (Fig. 4c, d).

There was no significant correlation between femoral MAP and CI_PFX_ before (*r*^2^ = 0.01, *p* = 0.09) and after (*r*^2^ = 0.001, *p* = 0.55) CPB.

## Discussion

Our study demonstrated that semi-invasive autocalibrated arterial waveform analysis was not able to reliably measure CI compared with TPTD before and after CPB. The investigated monitoring system did not seem to be affected by SVRI and MAP. With respect to haemodynamic changes, the semi-invasive device showed an acceptable trending capability before and after CPB.

The recently introduced semi-invasive PulsioFlex monitoring system was developed to determine beat-to-beat CI and other variables such as SVI and SVRI by pulse contour analysis. A less invasive and easy to install method without the need for calibration could be advantageous in daily clinical practice, since it may decrease the reluctance of physicians to use advanced haemodynamic monitoring [12]. In the past, estimation of CI was mostly performed by pulmonary thermodilution, a time consuming technique which requires experience and is frequently associated with method related complications. In this context, quick availability, simple installation, easy interpretation of estimated values and less invasiveness are advantages for monitoring systems based on pulse contour analysis.

Today, clinicians can choose between a wide variety of less-invasive or non-invasive devices based on pulse contour analysis. Each device consists on a proprietary software algorithm and most of them are based on the findings by Otto Frank [13]. However, several studies demonstrated a lack of accuracy for these monitoring systems in the presence of changing vascular tone [14, 15]. A recent meta-analysis dealing with five different pulse contour monitoring systems revealed an acceptable accuracy in presence of haemodynamic stable conditions. However, the authors found higher percentage errors and bias in haemodynamic unstable patients compared with thermodilution [10]. There are only very limited data concerning the accuracy of the new PulsioFlex monitoring system. A recently published study investigated the PulsioFlex system in patients undergoing off-pump coronary artery surgery [16]. The authors found an acceptable accuracy for the semi-invasive device compared with transpulmonary thermodilution. Bland-Altman analysis revealed a slight underestimation of CI by pulse contour analysis with low bias and small LOA`s. Overall percentage error was slightly above the 30 % limit which was recommended by Critchley and colleagues [9]. Our findings were in contrast to these results. We found poor accuracy for the PulsioFlex monitoring system in haemodynamic stable patients and observed percentage errors which clearly indicate no interchangeability with the reference technique, transpulmonary thermodilution. A possible explanation for these conflicting results could be the underlying software algorithm. Based on the PiCCO algorithm (as used currently in the PiCCO_2_), the PulsioFlex monitoring system uses a modified version of Wesseling`s cZ algorithm. This algorithm analyses the actual shape of the pressure waveform with the focus on the dicrotic notch. After calculation of the exponential decay time by analysing the pressure curve following the dicrotic notch, pressure related compliance can be computed. The area under the systolic portion of the arterial pressure waveform is also taken into account [17, 18]. Keeping in mind that pressure curves differ from central to peripheral arteries [19, 20] the PiCCO system usually consists on a femoral artery catheter. The tip of the transpulmonary thermodilution catheter is located in the abdominal aorta leading to more central pressure waveforms. In this context, it must be noted that the above mentioned study which observed sufficient accuracy for the new semi-invasive device used the femoral artery signal as input for pulse contour analysis [16]. Interestingly, a recently published study investigated the PulsioFlex monitoring system in critically ill patients with a femoral arterial line and observed unreliability for estimation of absolute CI values but reliability for tracking CI changes [21]. This is of high clinical importance, as these systems may be often used as “add on” by using existing radial artery catheters. Recent studies could demonstrate that the location of the arterial catheter plays a major role concerning the accuracy of uncalibrated pulse contour analysis [22]. The PulsioFlex monitoring system is provided with an autocalibration mode, which calculates the initial CI by using patient specific data and an unique unpublished algorithm. Thereafter, estimation of CI is performed by the well known PiCCO_2_ pulse contour algorithm [17].

Keeping in mind, that there is a mathematical coupling between SVRI and CI, we observed a weak but significant correlation between SVRI and CI by pulse contour analysis. To evaluate the effect of SVRI on the differences of CI between techniques, we calculated the correlation between the bias of CI_PFX_ and CI_TPTD_ and corresponding SVRI as suggested by recent literature [23]. We observed no significant correlation between the bias and SVRI before (*r* = −0.02, *p* = 0.79) and after (*r* = −0.07, *p* = 0.29) CPB. This result suggests that SVRI does not have a considerable impact on accuracy of CI_PFX_. In contrast, other studies, investigating an earlier PiCCO algorithm, showed an impairment of pulse contour analysis by changes in systemic vascular resistance [24]. However, we did not observe a significant relationship between CI by semi-invasive arterial waveform analysis and mean arterial pressure.

We studied this new monitoring system in patients undergoing elective coronary artery surgery under varying haemodynamic conditions, e.g. during a PLR manoeuvre before surgery. During the PLR manoeuvre, the semi-invasive monitoring system also failed interchangeability with the reference technique (CI_PFX_ PE 68 %). As suggested by recent literature [25], we calculated the precision of CI_TPTD_ and CI_PFX_ before and after CPB and found low coefficients of variation emphasizing our experience as we observed no rapid changes during data collection.

However, beside estimation of absolute values of CI, instantaneous tracking of haemodynamic trends could be extremely valuable for the clinician in the decision making process related to haemodynamic optimization. As recommended by recent literature, we excluded changes of CI obtained by transpulmonary thermodilution <15 % from further analysis [26]. Based on these criteria, concordance rates over 95 % indicate good trending ability, rates between 90–95 % are acceptable and concordance rates below 90 % should be considered as poor trending. The semi-invasive system overestimated absolute values of CI but showed an acceptable ability for following trends before and after CPB. It must be noted, however, that if exclusion zones were not applied, concordance rates in our study fell below 90 %. This could be explained by a central zone effect, respectively by the exclusion zone. Data points close to the center of the four quadrant plots represent small CI changes. These changes are most probably due to random error effects and therefore do not represent true CI changes. In addition, with respect to distribution of measurement errors, a precision of 20 % is considered as the upper limit of acceptance when using a reference method such as thermodilution. Therefore, exclusion of data points by an exclusion zone of 0.5–1.0 L/min or 15 % is considered as the gold standard. Interestingly, exclusion zones can be reduced to 5 and 10 % when an aortic flowprobe is applied as the reference technique [26]. With respect to trending ability, our findings were in agreement with other studies dealing with uncalibrated pulse contour analysis [27]. However, recent literature emphasized statistical limitations of four quadrant plots and recommended polar plots for trending analysis [28]. In our study, polar plot analysis revealed a poor trending ability before CPB and an acceptable trending of changes in CI after CPB for the investigated autocalibrated monitoring system. With respect to trending ability, a recent published investigation demonstrated poor trending for the new semi-invasive monitoring system in patients undergoing off-pump cardiac surgery [16]. Due to a small number of patients and possible random observation, the authors emphasized careful interpretation of their trending results. However, a recent study dealing with perioperative haemodynamic optimization by autocalibrated pulse contour analysis for CI trending was able to show a reduction in postoperative complications [29].

Some limitations must be emphasized in our study.

We excluded patients with haemodynamic instability, shock or lack of sinus rhythm and investigated patients undergoing elective coronary surgery with normal left ventricular function and without continuous high-dose (>0.1 μg/kg/min) catecholamine therapy. Therefore, our results cannot be extrapolated to patients with impaired left ventricular function, low cardiac output, cardiac arrhythmias or patients receiving continuous high-dose inotropic or vasoactive support.

Due to interferences of thermodilution curves caused by temperature fluctuations, transpulmonary thermodilution as the reference technique has some limitations especially after CPB. The probable reason for these transient thermal changes might be an influx of cold blood from hypoperfused compartments during CPB [30, 31]. In addition, accuracy of pulse contour analysis may also be affected by CPB. Recent investigations demonstrated abnormal aortic-to-radial arterial pressure gradients following CPB [32]. Future studies should investigate the reliability of this semi-invasive monitoring system in critically ill patients with both a femoral and a radial artery signal as input for pulse contour analysis.

## Conclusions

In conclusion, we observed poor accuracy for the new pulse contour monitoring system before and after CPB compared with transpulmonary thermodilution in patients undergoing coronary artery surgery. Furthermore, the semi-invasive device failed to meet criteria of interchangeability during a PLR-manoeuvre. There was a no significant influence of SVRI and MAP on CI by semi-invasive pulse contour analysis. However, we obtained an acceptable reliability for tracking changes of CI by the new semi-invasive monitoring system after CPB. Since we investigated only a homogeneous elective patient population, the present results cannot be readily transferred to other groups of patients.
